# Dihydroberberine exhibits synergistic effects with sunitinib on NSCLC NCI‐H460 cells by repressing MAP kinase pathways and inflammatory mediators

**DOI:** 10.1111/jcmm.13178

**Published:** 2017-04-26

**Authors:** Bingling Dai, Yujiao Ma, Wenjie Wang, Yingzhuan Zhan, Dongdong Zhang, Rui Liu, Yanmin Zhang

**Affiliations:** ^1^ School of Pharmacy Health Science Center Xi'an Jiaotong University Xi'an China

**Keywords:** dihydroberberine, sunitinib, combination therapy, NSCLC, MAPK, anti‐inflammation

## Abstract

Highly effective and attenuated dose schedules are good regimens for drug research and development. Combination chemotherapy is a good strategy in cancer therapy. We evaluated the antitumour effects of dihydroberberine combined with sunitinib (DCS) on the human non‐small cell lung cancer cell lines (NSCLC), A549, NCI‐H460, and NCI‐H1299 *in vitro* and *in vivo*. DCS showed synergic effects on NCI‐H460 cell proliferation, colony formation and transplantable tumour growth, which suggested dihydroberberine increases the sensitivity of lung carcinoma to sunitinib. Further studies indicated that DCS down‐regulated phosphorylation of JNK, p38, and NF‐κB in NCI‐H460 cells and tumours and suppressed the IκB and COX‐2 expression. In addition, DCS reduced the secretion of the pro‐inflammatory cytokine, interleukin‐1 (IL‐1), in tumours. Inhibition of p38 activation by DCS was a likely contributing factor in IL‐1 and COX‐2 down‐regulation. Consistent with these results, a genomewide microarray analysis found that DCS induced the expression of cell cycle signal molecules that are known to be affected by JNK and p38. The change of cell cycle, in turn, led to down‐regulation of JNK and p38, and further reduced IL‐1 secretion. Collectively, these findings highlight potential molecular mechanisms of DCS chemotherapeutic activity and suggest that DCS is an efficacious strategy in NSCLC therapy.

## Introduction

Lung cancer is one of the top three cancers and the leading cause of cancer mortality among men and women worldwide, with about 85% of cases diagnosed as non‐small cell lung cancer (NSCLC) 1, 2. The majority of patients with NSCLC are already in an advanced stage at diagnosis, and surgical treatment is not curative, so chemotherapy remains a mainstay of therapy 3. Therefore, novel chemotherapeutic agents are urgently needed. Combination therapy, which improves the curative effect and reduces the toxicity of these drugs, has become one of the most important means of improving survival of patients with lung cancer 4. In addition, agents with anti‐inflammatory activities may exert a chemoprotective effect on normal tissues against the toxic insults of chemotherapy.

Accumulating evidence has verified that many inflammatory signalling pathways are activated in various cancers; therefore, inflammation plays a critical role in multiple stages of tumour development, including initiation, promotion, invasion and metastasis 5, 6. Lung cancer is associated with an inflammatory environment, and many inflammatory mediators promote the development of lung cancer 7. Meanwhile, it has been reported that activation of the MAPK pathway plays an important role in the initiation and development of inflammatory processes. MAPK signalling cascades are important regulators of pro‐inflammatory cytokines 8, which transmit signals through sequential phosphorylation events. MAPKs can phosphorylate transcription factors or other downstream kinases that up‐regulate the expression of pro‐inflammatory mediators of extracellular stimuli 9. There are three major subfamilies of MAPK: the extracellular‐signal‐regulated kinases (ERK1/2), the c‐Jun N‐terminal or stress‐activated protein kinases (JNK or SAPK), and p38 MAPKs 10, 11, 12. Among mediators, three prominent cytokines, that is tumour necrosis factor alpha (TNF‐α), interleukin‐6 (IL‐6) and interleukin‐1 (IL‐1), could contribute to the development of lung cancer 13. These cytokines could induce transcription factors that drive inflammation, including NF‐κB and STAT3. NF‐κB, which is one of many downstream targets of TNF receptor 1 activation, is an important, ubiquitous transcription factor known to regulate the expression of genes involved in inflammation, cell proliferation, migration, extracellular matrix turnover and angiogenesis 14, 15.

**Figure 1:**
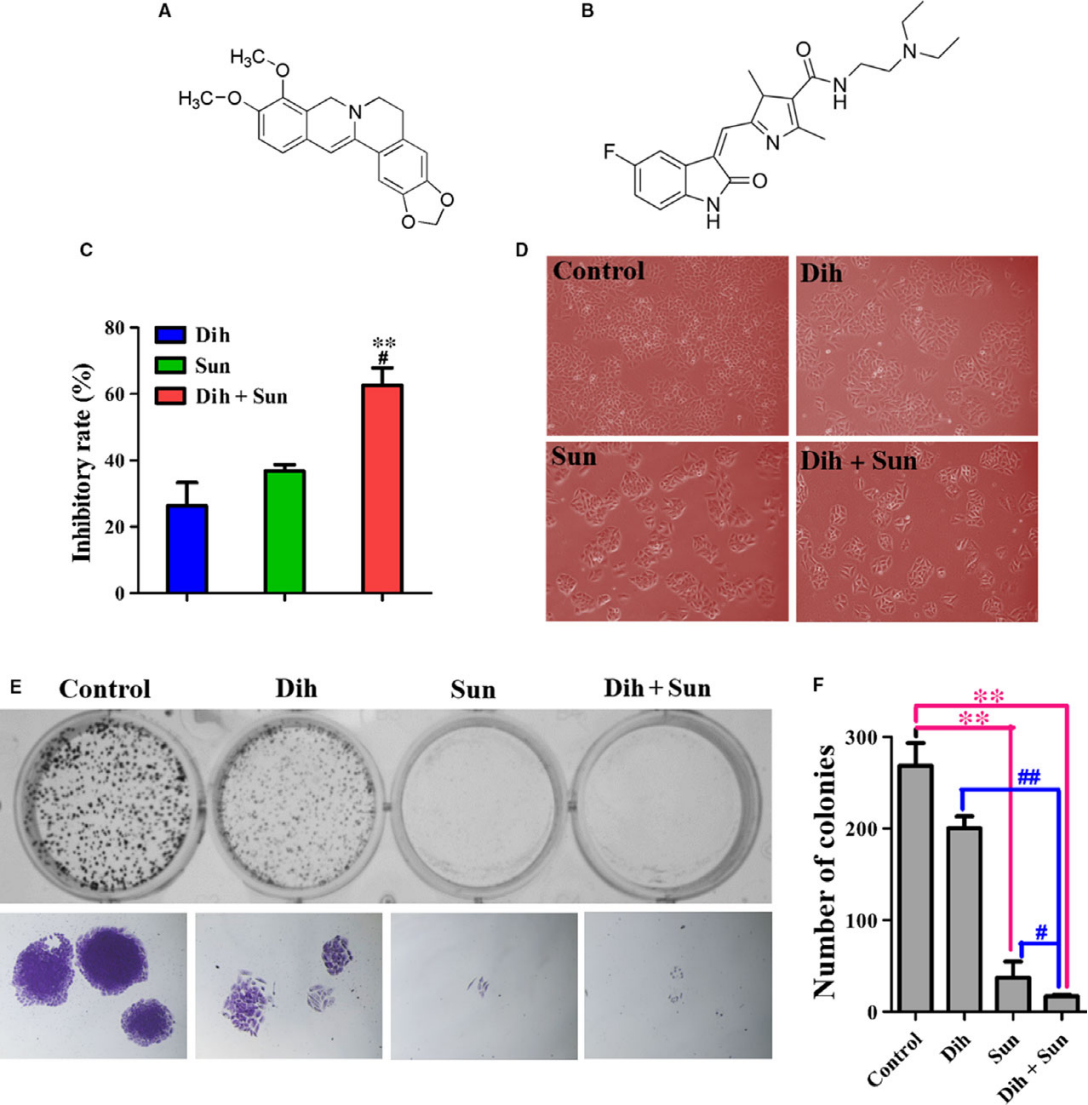
Effects of dihydroberberine and/or sunitinib on the growth of lung cancer cells. (**A**) Chemical structure of dihydroberberine. (**B**) Chemical structure of sunitinib. (**C**) NCI‐H460 cells were treated with dihydroberberine, sunitinib or DCS for 48 hrs. **P < 0.01 compared to dihydroberberine‐treated group; ^#^ *P* < 0.05 compared to sunitinib‐treated group. (**D**) Effect of dihydroberberine and/or sunitinib on NCI‐H460 cell morphology. (**E**) Effect of dihydroberberine and/or sunitinib on the colony formation of NCI‐H460 cells. The top row depicts colony formation, and the bottom row expresses the individual colony. (**F**) Quantification of the number of colonies following treatment of the cells with dihydroberberine, sunitinib or DCS. Values are presented as mean ± SEM (*n* = 3). **P < 0.01 compared to control group. ^#^ *P* < 0.05, ^##^ *P* < 0.01 compared to Dih‐treated and Sun‐treated group.

*Berberis* is a traditional Chinese medicine. Berberine is an alkaloid that is isolated from *Berberis* having biological and pharmacological activities, including antibacterial, anti‐inflammatory, and anticancer activities, and has been widely used in human health care 16. However, there have been few reports of berberine's analogue, dihydroberberine (Fig. 1A), especially with regard to its antitumour activity. Sunitinib (Fig. 1B) is a multitargeted receptor tyrosine kinase inhibitor that is effective against many tyrosine kinases, including PDGFR, VEGFR, FLT‐3, CSF‐1R, kit and ret. Preclinical and clinic trials have demonstrated that sunitinib has potent antitumour activity against renal cell carcinomas, gastrointestinal stromal tumours, breast carcinomas, NSCLC and other cancers. Its anticancer activity results from a dual inhibitory effect towards angiogenesis and tumour cell proliferation. Its main adverse reactions include hypodynamia, nausea and thrombocytopenia 17, which can be attributed to megadosing. In our study, we explored the effect of dihydroberberine on lung cancer cells and investigated the synergistic action of dihydroberberine and sunitinib on NCI‐H460 lung carcinoma cells *in vitro* and *in vivo*. Here, we report our novel findings that dihydroberberine decreased the effective dose of and increased the sensitivity of lung carcinomas to sunitinib.

## Materials and methods

### Cell lines and mice

Human lung cancer cell lines A549 (TCHu150), NCI‐H1299 (TCHu160) and NCI‐H460 (TCHu205) were purchased from the Shanghai Institute of Cell Biology in the Chinese Academy of Sciences (SH cellbank). These cell lines were frozen immediately after receipt from SH cellbank, and resuscitated in RPMI 1640 media (Sigma‐Aldrich, St. Louis, MO, USA), supplemented with 10% foetal bovine serum (HyClone, Logan, Utah, USA), before use. Four‐ to six‐week‐old ALB/C nude male mice were purchased from Hunan SJA Laboratory Animal Co., Ltd. (Hunan, PR. China) and housed in the Experimental Animal Center of Xi'an Jiaotong University. The entire procedure was carried out in accordance with the approved guidelines of the regional authorities, according to China animal care regulations.

### Statement identifying the institutional and/or licensing committee experimental approval

All animal experiments were carried out according to the guidelines and approval of the Institutional Animal Care and Use Committee of Xi'an Jiaotong University.

### Cell proliferation assay and synergistic screening

For drug toxicity assays, cells (A549: 5 × 10^3^ cells/well, NCI‐H1299: 1 × 10^4^ cells/well and NCI‐H460: 1 × 10^4^ cells/well) were seeded in 96‐well plates. The following day, the indicated concentrations of dihydroberberine and sunitinib (dissolved in serum‐free media) were added to the plates for 48 hrs. Cell viability was assessed using the MTT assay, and absorbance was measured at 490 nm with a microplate reader (Bio‐Rad, Hercules, CA, USA).

To determine the combination index (CI), different concentrations of dihydroberberine and sunitinib were added to adherent NCI‐H460 cells for 48 hrs, and cell viability was calculated, as indicated above. The CI, which is a parameter that indicates whether the interaction of 2 or more drugs is synergistic, additive or antagonistic, was calculated using the following equation: *Q* = *E*_AB_/(*E*_A_ + *E*_B_ − *E*_A_ *E*_B_). (*E*_AB_: the inhibition rate of drug A plus drug B; *E*_A_: the inhibition rate of drug A; *E*_B_: the inhibition rate of drug B).

### Colony formation assay

Exponentially growing NCI‐H460 cells were seeded in 12‐well plates (300 cells/well) and treated with 25 μmol/l dihydroberberine, 2 μmol/l sunitinib, or 25 μmol/l dihydroberberine plus 2 μmol/l sunitinib for 48 hrs. Subsequently, the media was changed, and the cells were cultured in drug‐free media for an additional 10–15 days, until colonies were obviously visible and countable. Then, the colonies were fixed with methanol and stained with crystal violet. Images were photographed under the chemiluminescent and fluorescent imaging system (Champchemi Professional, SG2010084, Sage creation, Beijing, China) and an inverted fluorescence microscope (DM505, Nikon Co., Ltd., Otawara, Tochigi, Japan).

### *In vivo* tumour suppression assay

Four to six‐week‐old non‐obese diabetic severe combined immunodeficiency (NOD/SCID) mice were injected subcutaneously into the right flank with 4 × 10^6^ NCI‐H460 cells suspended in sterile physiological saline. Each tumour was measured by calliper every other day, and its volume was calculated using the formula: volume = (length × width^2^)/2. Studies were initiated when tumour volume reached 80–100 cm^3^. Mice were randomly assigned to four groups (five mice/group) and treated with vehicle (0.5% CMC‐Na), sunitinib (20 mg/kg in 0.5% CMC‐Na) once daily, dihydroberberine (250 mg/kg in 0.5% CMC‐Na) or dihydroberberine plus sunitinib (250 mg/kg dihydroberberine + 20 mg/kg sunitinib in 0.5% CMC‐Na) every other day by intragastric administration. Mouse weight and tumour volume were monitored every other day. After 14 days, mice were killed, and the tumours were frozen at −80°C for Western blot analysis and fixed in 4% paraformaldehyde for immunohistochemical analysis.

### HE staining and immunohistochemistry (IHC)

Tumour specimens were embedded in paraffin and cut into 4 μm‐thick sections for HE staining and IHC. The SV histostain kit (Boster bioengineering Co. LTD, Wuhan, China) was used for IHC, according to the manufacturer's instructions. The antibodies used in IHC were anti‐ki67 (1:80 dilution), anti‐COX‐2 (1:100 dilution), anti‐NF‐κB p65 (1:100 dilution), anti‐JNK2 (1:150 dilution), anti‐phospho‐JNK (1:150 dilution), anti‐phospho‐p38 (1:100 dilution) and anti‐p38 (1:50 dilution).

### Antibodies and Western blotting

For Western blotting, proteins were extracted by lysing cells and frozen tissue from nude mice in ice‐cold RIPA lysis buffer that contained protease inhibitors and phosphatase inhibitors (Roche, Nutley, NJ, USA). Protein was quantified using the BCA assay (Pierce Biotechnology, Rockford, Illinois, USA). Fifty micrograms of total protein per lane was resolved using 10% SDS‐PAGE gels and then transferred to polyvinylidene fluoride membranes. Membranes were probed with primary antibodies. Following incubation with horseradish peroxidase‐conjugated secondary species‐specific antibodies (Pierce Biotechnology), immunoreactive proteins were detected by enhanced chemiluminescent (ECL) plus reagent (Pierce Biotechnology). Gels were run under the same experimental conditions, and GAPDH was used as a loading control. Cropped gel images are shown in the Figures, and the grey‐scale values of bands were analysed using Image Pro Plus software (Image‐Pro Plus 5.1, Media Cybernetics, Inc., Rockville, MD, USA). Target protein expression was calculated as the ratio of grey scanning values.

### Elisa

Protein extracted from frozen tissue was quantified by BCA reagent (Pierce Biotechnology), and 200 μg of total protein was used to determine the levels of TNF, IL‐1α and IL‐6α by commercially available ELISA kits (Neobioscience Technology Company, Shenzhen, China). Protocols were performed according to the manufacturer's instructions.

### Microarray analysis

NCI‐H460 cells were treated with various combinations of dihydroberberine and sunitinib for 48 hrs. Total RNA was extracted with TRIzol (Invitrogen, USA) reagent at room temperature and then stored at −80°C. Microarray experiments were performed with a Whole Human Genome Oligo Microarray (Affymetrix GeneChip PrimeView Human Gene Expression Array, Santa Clara, CA, USA), which contained more than 49,000 human genes and transcripts. The entire procedure was conducted at the Shanghai Biotechnology Corporation, China. Arrays were scanned by Affymetrix GeneChip^®^ Scanner 3000 (Cat#00‐00213, Affymetrix, Santa Clara, CA, USA). Command Console Software (Affymetrix, Santa Clara, CA, USA) was used to control the scanner and summarize probe cell intensity data (CEL file generation) with default settings. Raw data were normalized by Expression Console, and, after GO annotation, genes with ≥ twofold differences between groups were determined to be statistically significant if *P* ≤ 0.01. All microarray data sets were submitted to the ‘Gene Expression Omnibus’ database with an accession number of GSE70282.

### Analyses of the cell cycle and cell apoptosis

Exponentially growing NCI‐H460 cells were serum starved for 24 hrs. After co‐culture with 25 μmol/l dihydroberberine, 2 μmol/l sunitinib, or 25 μmol/l dihydroberberine plus 2 μmol/l sunitinib for 48 hrs, cells were harvested, washed with PBS and suspended in 70 % ice‐cold ethanol solution and incubated at −20°C overnight. After fixation, the cells were washed thrice with PBS and incubated with 1 ml RNase (50 μg/ml) and 1 ml PI (60 μg/ml) for 30 min in the dark at room temperature.

Cells treated with 25 μmol/l dihydroberberine, 2 μmol/l sunitinib, or 25 μmol/l dihydroberberine plus 2 μmol/l sunitinib for 48 hrs were harvested and washed with PBS. After centrifugation, cells were suspended in binding buffer and incubated with 5 μl AnnexinV‐FITC. 3 min later, 10 μl PI (20 μg/ml) was added and incubated in the dark at room temperature for 10 min.

All stained cells were analysed by FACS (Becton Dickinson, Mountain View, CA, USA). The obtained data were analysed with Modfit LT software.

### Statistical analyses

All data are expressed as mean ± S.E.M in quantitative experiments. Statistical analyses of the differences between groups were performed with ANOVA. A *P*‐value of less than 0.05 was considered statistically significant.

## Results

### Individual effects of dihydroberberine and sunitinib on lung cancer cells

To determine the effects of dihydroberberine and sunitinib on NSCLC cells, dose–response curves were constructed. After 48 hrs of drug exposure, the growth of A549, NCI‐H460 and NCI‐H1299 cells was significantly inhibited in a dose‐dependent manner *in vitro* (Fig. S1A and B). The IC50 values of dihydroberberine on A549, NCI‐H460, and NCI‐H1299 cells were 11.17, 46.33 and 37.91 μmol/l, respectively. In addition, the IC50 values of sunitinib on A549 cells, NCI‐H460 cells, and NCI‐H1299 cells were 2.04, 4.03 and 5.45 μmol/l, respectively. Dihydroberberine and sunitinib showed less growth suppression of NCI‐H460 cells than A549 and NCI‐H1299 cells. In order to increase the sensitivity of NCI‐H460 cells to dihydroberberine and sunitinib, we explored the effect of simultaneous treatment (dihydroberberine combined with sunitinib, DCS).

### Synergistic effects of DCS on NCI‐H460 cell proliferation and colony formation

To determine the synergistic effects of DCS on NCI‐H460 cells, DCS at different concentrations was studied by the method of Chou *et al*. 18. The inhibition rate of dihydroberberine (0, 6.25, 12.5, 25 μmol/l) combined with sunitinib at different concentrations (0, 1, 2, 4 μmol/l) on the growth of NCI‐H460 cells is shown in Figure S2, and the CIs of DCS are shown in Table S1. As shown in Figure 1C and D, 25 μmol/l dihydroberberine, 2 μmol/l sunitinib, and their combination had inhibition rates of 26.32%, 36.78% and 62.62%, respectively, in NCI‐H460 cells. Meanwhile, the CI value of 25 μmol/l dihydroberberine combined with 2 μmol/l sunitinib was 1.17, which indicated a better synergistic effect of inhibiting NCI‐H460 cell viability than the individual compounds. The colony formation assay showed that the colonies treated with DCS were significantly fewer in number and smaller in size than the control cells and Dih‐/Sun‐treated cells (Fig. 1E and F), which further confirmed the synergistic effects of DCS.

### Antitumour efficacy of DCS *in vivo*

**Figure 2:**
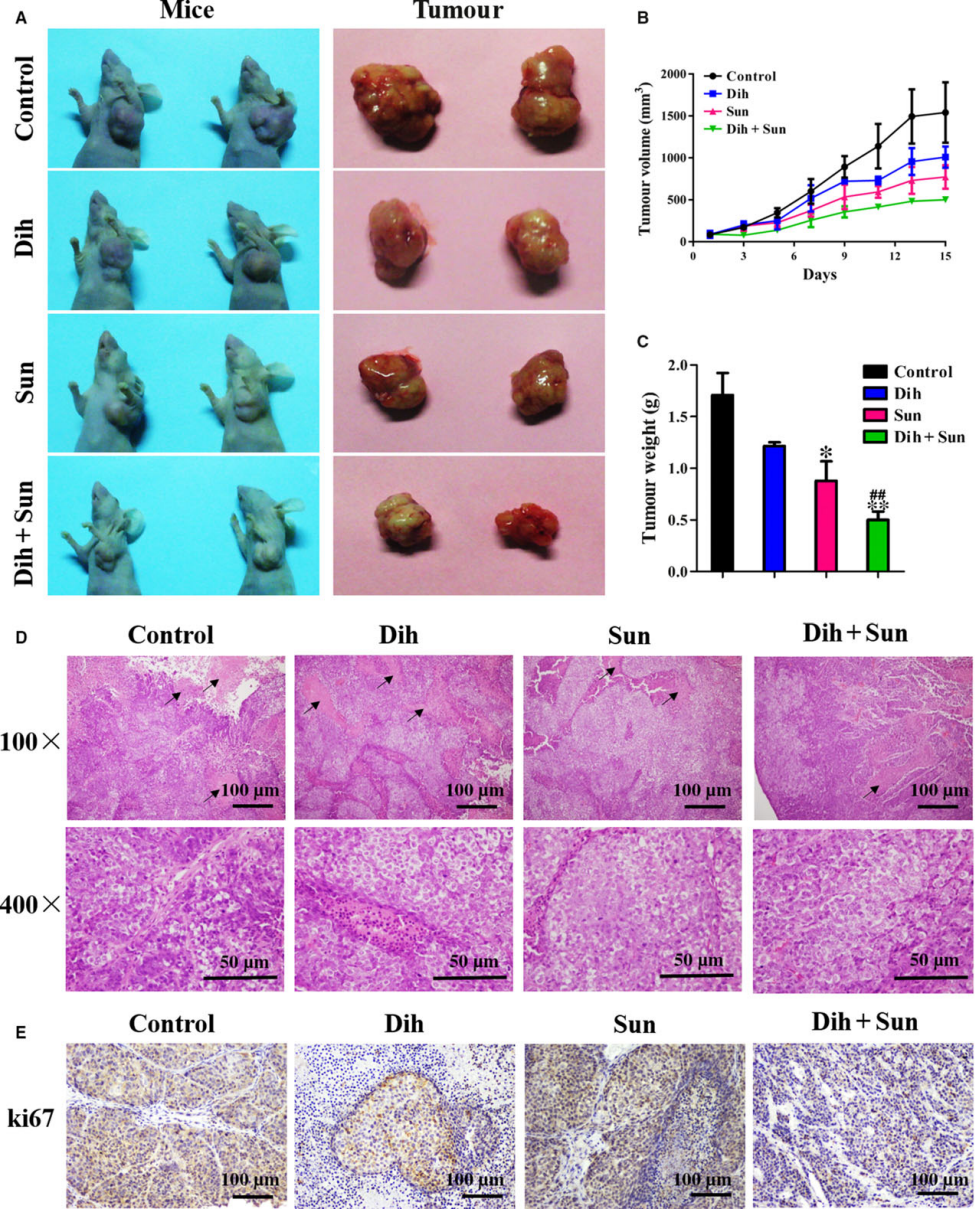
Effects of DCS on the growth of NCI‐H460 xenografts. Mice bearing NCI‐H460 tumours were treated with vehicle daily, sunitinib (20 mg/kg) daily, dihydroberberine (250 mg/kg) every other day or DCS every other day by intragastric administration. (**A**) Representative xenograft tumours of NCI‐H460 human lung cancer in each group. (**B**) Mean tumour volume ± SEM at a given time‐point. (**C**) Mean tumour weight at killing. *P < 0.05, **P < 0.01 compared to control group; ^##^ *P* < 0.01 compared to dihydroberberine‐ or sunitinib‐treated groups. (**D**) Necrosis and mitosis observed in each group using HE staining. (**E**) Representative images of ki67 staining in each group by immunohistochemistry.

To evaluate the effect of DCS on tumour growth *in vivo*, a xenograft tumour model in nude mice was established. The anticancer effects of DCS against NCI‐H460 transplanted tumours are shown in Figure 2A. Tumour volume was recorded every 2 days and is shown in Figure 2B. On day 15, all of the mice were killed, and tumours were excised and weighed (Fig. 2C). These results show that the average tumour weight with vehicle, dihydroberberine, and sunitinib treatment groups was 1.71 ± 0.43, 1.22 ± 0.05 and 0.88 ± 0.38 g, respectively. For comparison, the tumour weight with DCS was 0.47 ± 0.07 g. Therefore, while 250 mg/kg dihydroberberine and 20 mg/kg sunitinib inhibited NCI‐H460 xenograft tumour growth by 28.65% and 48.53%, respectively, the combination of dihydroberberine and sunitinib had a marked inhibitory effect of 72.51%. Furthermore, there were no significant changes in the body weight of the mice during the experiment. The final average body weights were 23.64 ± 2.59, 23.11 ± 1.67, 25.59 ± 2.41, and 23.42 ± 1.56 g in control, dihydroberberine, sunitinib and DCS groups, respectively. The average body weights of the sunitinib‐treated mice increased slightly, but the differences were not statistically significant.

The H/E staining (Fig. 2D) shows large areas of necrosis and more mitosis in the tissue of the control group, which indicates that the tumour cells had grown quickly and caused necrosis. The area of necrosis in the dihydroberberine‐ and sunitinib‐treated groups was reduced compared to the control group, while a much more significant inhibition appeared in the combination group. Next, the effect of DCS on tumour growth was analysed by proliferation index ki67 staining of tumour biopsies obtained from each group of mice. The proliferation rates of tumours from mice treated with DCS were significantly lower compared to those from mice treated with vehicle, dihydroberberine or sunitinib (Fig. 2E). The data show that the effect of the combined treatment was superior to the effect of dihydroberberine or sunitinib individually. Collectively, these results validate that DCS has a good synergistic inhibition on tumour growth *in vivo*.

### Effect of DCS on PI3K/AKT/mTOR signalling cascades

To understand how DCS modulates various cell signalling pathways, the effects of DCS on PI3K/AKT/mTOR signalling cascades were analysed. As shown in Figure S3A, B, and C, DCS had no effect on the protein levels of PI3Kp110α, PI3Kp110β, PI3Kp110γ and PI3Kp110III subunits. There was also no additional effect of DCS on the phosphorylation of PI3Kp85/p55 in NCI‐H460 cells compared with dihydroberberine or sunitinib alone. In addition, signalling of PI3K through the downstream effectors, AKT and mTOR, did not show obvious changes in NCI‐H460 cells and tumour tissues (Fig. S3D, E and F).

### Effect of DCS on the MAPK signalling pathway

**Figure 3:**
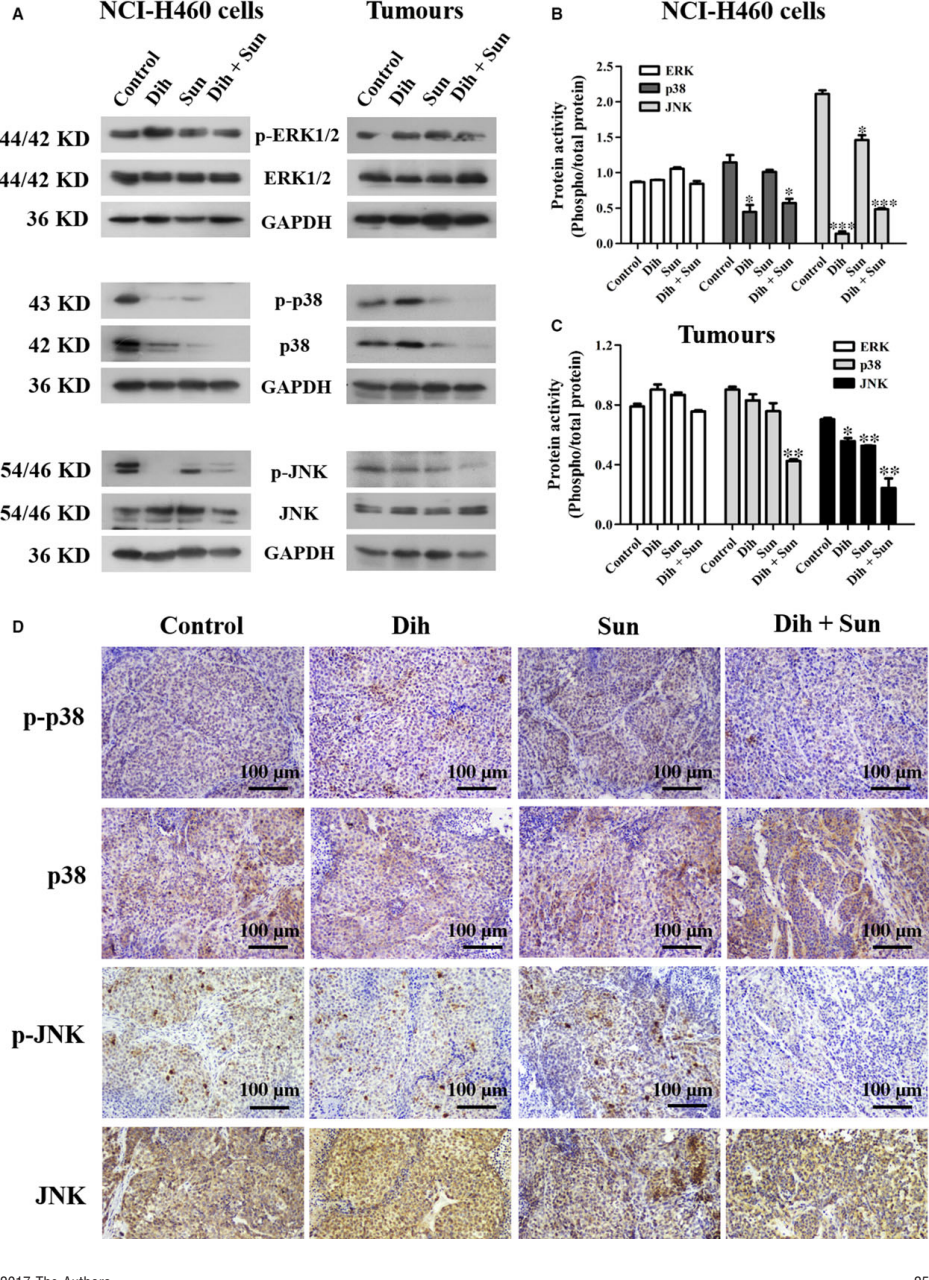
DCS‐regulated MAPK signalling pathway proteins in NCI‐H460 cells. (**A**) Effect of DCS on MAPK signal protein expression of p‐ERK, ERK, p‐p38, p38, p‐JNK and JNK in NCI‐H460 cells and tumour tissues using Western blotting. (**B**,** C**) Quantification of the data from part (**A**). DCS decreased the phosphorylation of p38 and JNK, but had no effect on ERK. *P < 0.05, **P < 0.01, ***P < 0.001, compared to control group. (**D**) p38 and JNK immunohistochemical staining in tumour tissues of NCI‐H460 cells harvested from xenograft mice (100 × magnification).

To clarify the underlying inhibition mechanism of DCS, we examined the effect of DCS on the MAPK cascades ERK, JNK, and p38 in NCI‐H460 cells and tumour tissues. As shown in Figure 3A, B, and C, the phosphorylation of JNK, phosphorylation of p38 MAPK and total p38 MAPK protein were decreased in the DCS groups compared with single agents in NCI‐H460 cells and tumours. By contrast, DCS had no effect on the phosphorylation of ERK in NCI‐H460 cells and tumours. The protein expression of JNK and p38 in tumours was detected by immunohistochemical staining (Fig. 3D), which provided *in vivo* evidence of the effect of DCS on MAPK signalling pathways. Consistent with the Western blot analysis, these results showed that DCS decreased the phosphorylation of JNK and p38 protein expression in xenograft tumours (Fig. 3D). Our data obviously indicate that DCS modulates JNK and p38 MAPK signal pathways, possibly inhibiting the proliferation of NCI‐H460 cells.

### Effect of DCS on inflammatory mediators

**Figure 4:**
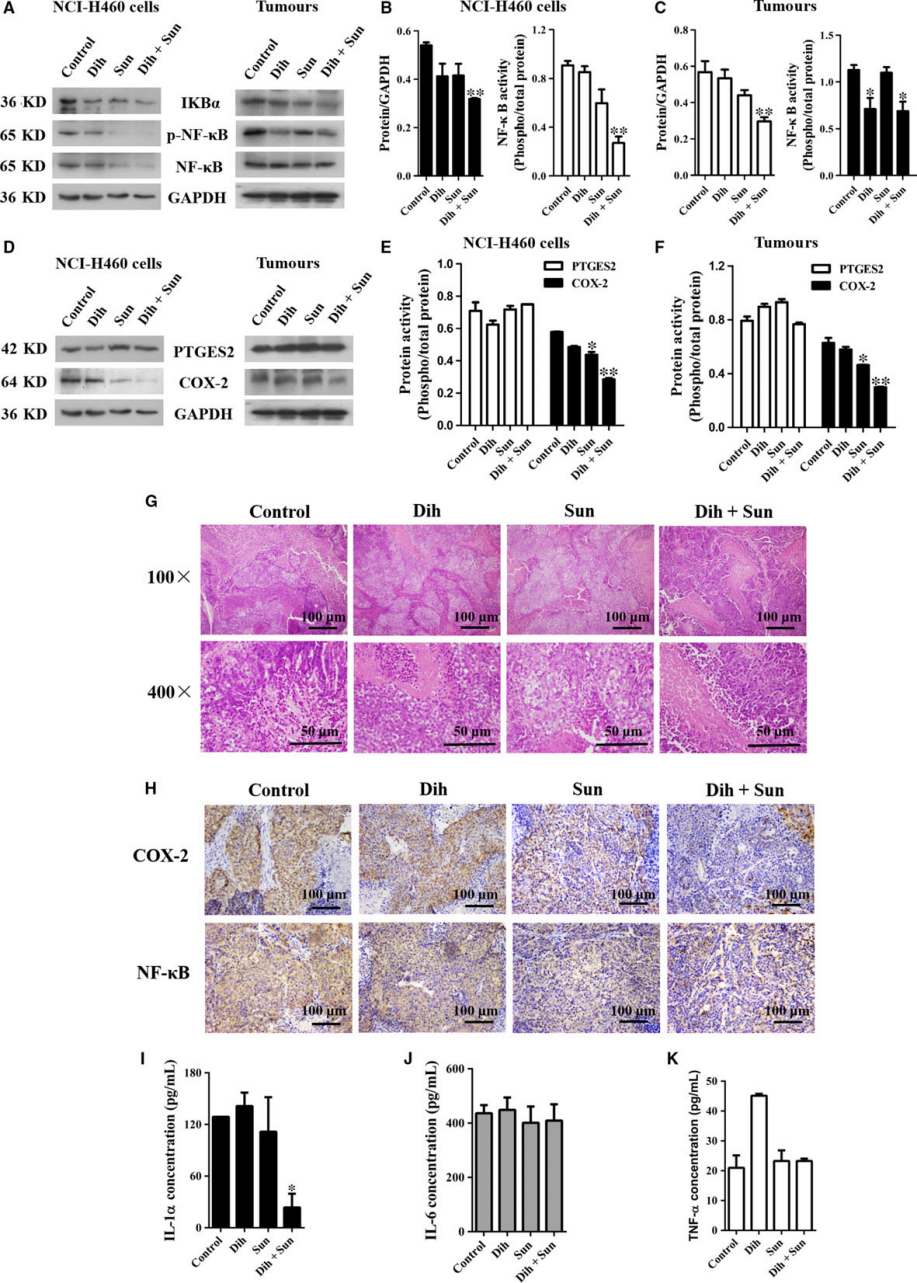
Effects of DCS on inflammatory mediators. (**A**) Effect of DCS on inflammation‐related protein expression of IKBα, p‐NF‐κB, and NF‐κB in NCI‐H460 cells and tumour tissues using Western blotting. (**B**,** C**) Quantification of the data from part (**A**). DCS decreased the expressions of IKBα and the phosphorylation of NF‐κB. (**D**) Effect of DCS on inflammation‐related protein expression of PTGES2 and COX‐2 in NCI‐H460 cells and tumour tissues using Western blotting. (**E**,** F**) Quantification of the data from part (**D**). DCS decreased the expression of COX‐2, but had no effect on PTGES2. *P < 0.05, **P < 0.01 compared to control group. (**G**) Inflammatory cells in tumour tissues, identified using HE staining. (**H**) COX‐2 and NF‐κB protein expression in tumour tissues of NCI‐H460 cells harvested from xenograft mice was stained by immunohistochemistry (100 × magnification). (**I**–**K**) IL‐1, IL‐6 and TNF‐α expression were assessed by ELISA in tissues excised from xenograft mice that were treated with dihydroberberine, sunitinib or DCS.

The H/E staining in Figure 4G shows fewer inflammatory cells in the DCS‐treated group. To further investigate the effects of DCS on inflammation, we measured inflammation‐related protein expression of IKBα, p‐NF‐κB, NF‐κB, prostaglandin E synthase 2 (PTGES2) and cyclooxygenase‐2 (COX‐2), in NCI‐H460 cells and tumour tissue. As shown in Figure 4A, B and C, DCS inhibited NF‐κB activation by blocking IκBα degradation. Moreover, DCS markedly down‐regulated the expression of the pro‐inflammatory protein, COX‐2, but had no effect on inhibition of PTGES2 (Fig. 4D, E and F). Meanwhile, immunohistochemistry results showed that DCS effectively inhibited the protein expression of COX‐2 and NF‐κB (Fig. 4H).

The levels of IL‐1, TNF‐α and IL‐6 in the tumour were also detected. Interestingly, as demonstrated in Figure 4I, J, and K, DCS effectively inhibited the production of IL‐1, but had no effect on TNF‐α and IL‐6. These results indicate that DCS diminishes pro‐inflammatory mediators by decreasing the expression of NF‐κB and subsequent pro‐inflammatory cytokine production.

### Differentially expressed genes with DCS

**Figure 5:**
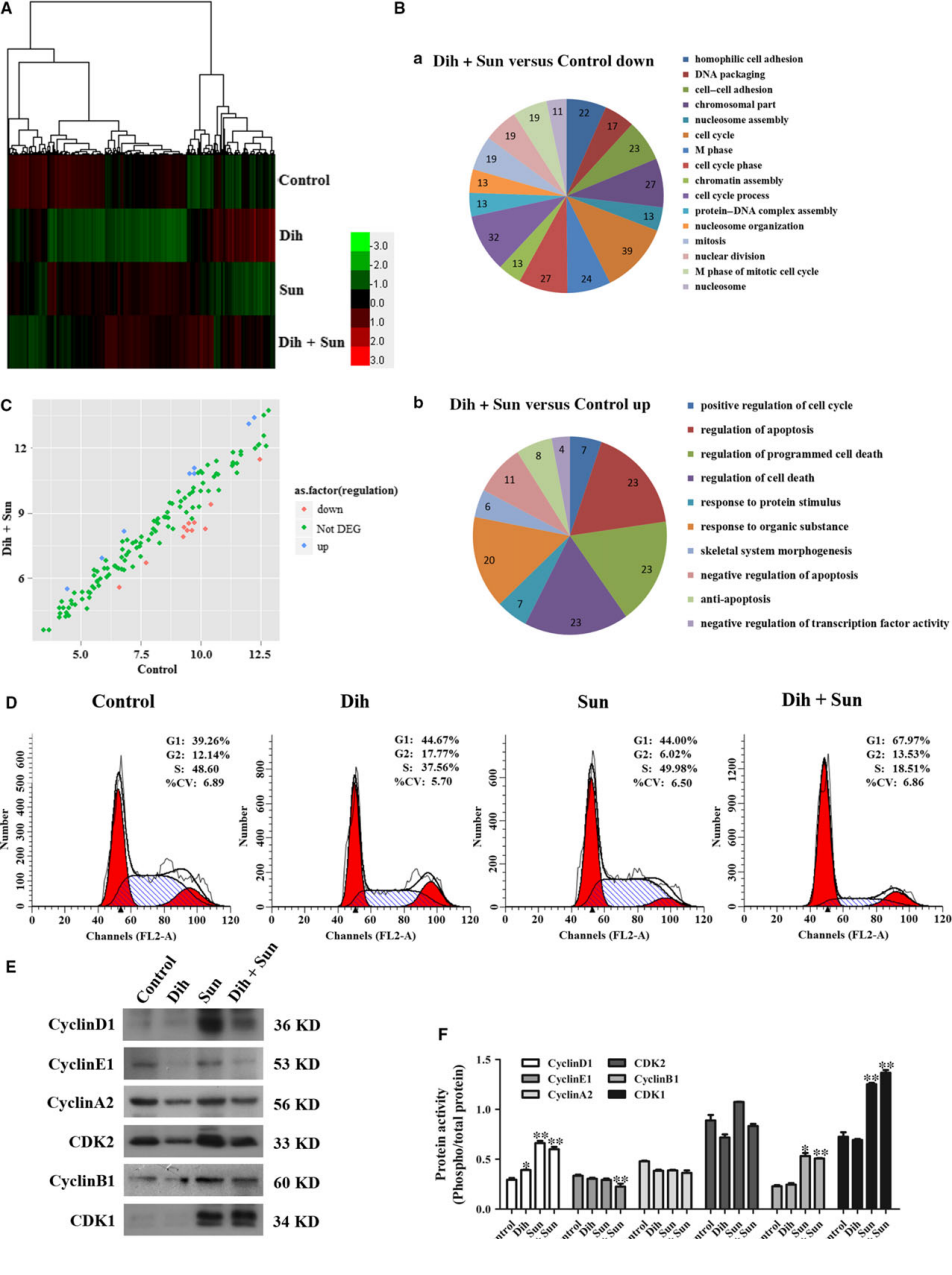
Effect of DCS on expression of genes according to functional classification. (**A**) Hierarchical cluster analysis of total genes among groups. Each column represents one sample, and each row depicts one gene. Red denotes an increase in gene expression, and green denotes a decrease in gene expression compared with the other groups. A brighter colour represents a greater gene expression difference. (**B**) The most significantly enriched pathways were plotted for pathway analysis of the identified genes. (**C**) Analysis of differentially expressed genes for cell cycle between the control and DCS‐treated groups. Each plot represents one gene. (**D**) Effect of 12k on NCI‐H460 cell cycle. The cell cycle progression was evaluated by FACS. (**E**) Effect of DCS on expression of the cell cycle signal proteins cyclin D1, cyclin E1, cyclin A2, CDK2, cyclin B1 and CDK1 in NCI‐H460 cells using Western blotting. (**F**) Quantification of the data from part (**D**). DCS decreased the amount of cyclin E1, up‐regulated the expression of cyclin D1, cyclin B1, and CDK1, but had no effect on cyclin A2 and CDK2. *P < 0.05, **P < 0.01 compared to control group.

To gain insights into the mechanism of DCS synergistic inhibition, we used an Affymetrix GeneChip PrimeView Human Gene Expression Array to compare mRNA expression levels between untreated cells and dihydroberberine‐ and/or sunitinib‐treated cells (Fig. 5A). In total, 1634 differentially expressed genes (495 up‐regulated and 1139 down‐regulated genes) were identified between control and DCS‐treated cells at *P* < 0.05 and fold‐change ≥2. A group of differentially expressed genes obtained from the primary analysis were analysed further by GO enrichment and pathway analysis. The down‐regulated genes and up‐regulated genes between control and dihydroberberine‐ or sunitinib‐treated groups are shown in Figure S4. Microarray results indicated that a large number of the top‐ranking, down‐regulated genes in DCS‐treated cells were associated with several cancer biological processes, such as cell cycle phase, cell cycle process, M phase, M phase of mitotic cell cycle, homophilic cell adhesion and cell–cell adhesion (Fig. 5B upper panel). As shown in the lower panel of Figure 5B, the main GO categories for up‐regulated genes in DCS‐treated cells included positive regulation of cell cycle, regulation of apoptosis, p53 signalling pathway and negative regulation of apoptosis. Among the differentially expressed genes were cell cycle‐related genes, as illustrated in Figure 5C.

### Effect of DCS on cell cycle and related proteins derived from gene expression array

To corroborate the results of the microarray analyses, we further explored the effect of DCS on cell cycle and the expression of cell cycle proteins that are closely related to the JNK and p38 MAPK signalling pathways. As shown in Figure 5D, as compared with controls, treatment of DCS induced an accumulation of NCI‐H460 cells in the G1 phase of the cell cycle. The population of untreated cells in G1 phase were 39.26%, whereas in cells treated with dihydroberberine, sunitinib and DCS, the population of cells in G1 phase showed a dose‐dependent increase and were 44.67%, 44.00% and 67.97%, respectively. A decrease in the population of cells in the S phase was also observed, and changed from 48.60% to 37.56%, 49.98% and 18.51%. Consistent with the microarray data, DCS significantly up‐regulated the protein levels of cyclin D1, cyclin B1, and CDK1, as well as down‐regulated the level of cyclin E1 compared to control; the expression of cyclin A2 and CDK2 did not change (Fig. 5E and F). These results indicate that DCS would induce an accumulation of NCI‐H460 cells in the G1 phase of the cell cycle. Our findings suggest that DCS inhibits NCI‐H460 cell viability and arrests the cell cycle at G1 phase by modulating JNK and p38 MAPK signalling.

### Effect of DCS on cell apoptosis

We also tested the effect of DCS on NCI‐H460 cell apoptosis. As shown in Figure S5, the FACS results showed that the early apoptotic cells (lower right) in the control group were 8.32%, after treatment with dihydroberberine, sunitinib and DCS, the percentage of early apoptotic cells were 13.62%, 42.38% and 56.46%, respectively. While there was few change in the late apoptotic cells (upper right). Our data suggest that DCS could induce apoptosis in NCI‐H460 cells.

## Discussion

Sunitinib is widely used in clinical treatment for several human malignancies. As it has undesirable side‐effects, sunitinib has been used in combination therapy, which results in superior antitumour activities and fewer side‐effects. Therefore, it is necessary to search for novel agents to enhance the anticancer effects of sunitinib. Berberine has been reported to have various pharmacological activities, but there have been no reports on its analogue, dihydroberberine. In the current study, we evaluated the effects of dihydroberberine on lung cancer cells, including NCI‐H460, A549, and NCI‐H1299 cells, and confirmed that dihydroberberine treatment resulted in a reduction of cell viability in a dose‐dependent manner. Meanwhile, we found that dihydroberberine combined with sunitinib at appropriate concentrations (dihydroberberine was 25 μmol/l and sunitinib was 2 μmol/l) had obvious synergistic inhibition on NCI‐H460 cell viability, compared with dihydroberberine or sunitinib alone. Furthermore, dihydroberberine combined with sunitinib displayed good inhibition on NCI‐H460 cell colony formation *in vitro* and xenografts in nude mice *in vivo*. Based on these promising results, we further explored the molecular mechanisms of the synergetic interaction of dihydroberberine and sunitinib.

The PI3K/AKT/mTOR signal‐transduction pathway plays a significant role in the progression of human lung cancer 19. In cancer, PI3K/AKT/mTOR is constitutively activated by PI3Ks (composed of p110 catalytic subunits and regulatory subunits) that catalyse phosphorylation to coordinate cell growth, cell migration and cell survival 20, 21. The MAPK signalling pathway belongs to a large family of serine–threonine kinases, which include ERK1/2, p38 and JNK, and forms major cell proliferation signalling pathways from the cell surface to the nucleus 22. ERK is activated by mitogenic stimuli, such as growth factors, cytokines, and phorbol esters, which are involved in the regulation of cell proliferation, differentiation and meiosis. The JNK family members are key molecules in the signal transduction of various kinds of stress, as well as in neural development, inflammation and apoptosis. They are activated by radiation, osmotic pressure, temperature and others factors 23. P38 mediates growth, inflammation and apoptosis; it has thus become a target in the development of anti‐inflammatory drugs 24. JNK and p38 are important regulators of inflammatory mediators, so the activation of MAPK pathways plays an essential role in the initiation and development of inflammatory processes. Our results indicate that dihydroberberine combined with sunitinib has no effect on various isoforms of PI3K, either the catalytic or regulatory subunits, and also has no effect on the downstream effectors, AKT and mTOR. Interestingly, we found that dihydroberberine combined with sunitinib inhibited JNK and p38 activation in NCI‐H460 cells and tumours, but had no effect on ERK. Therefore, our results indicate that DCS may be a good multitarget combinatorial drug.

p38 MAPKs are important members of the MAPK family in controlling inflammation, and they can be activated by numerous physiological and chemical stresses, osmotic stress, heat shock, UV irradiation, ischaemia and inflammatory stimuli (*e.g*. IL‐1, TNF and platelet‐activating factor) 25. The p38 MAPKs phosphorylate and activate several important transcription factors, including NF‐κB, p53 and ATF, which leads to the induction and release of pro‐inflammatory cytokines 26. NF‐κB is an important signal pathway, which plays a vital role in regulating the expression of multiple genes, including pro‐inflammatory cytokines (*e.g*. IL‐1, IL‐6, IL‐8, IL‐12, TNF‐α and interferon‐γ) 27. Increasing evidence reveals that the inhibition of NF‐κB activity may lead to alleviating the severity of inflammatory diseases 28. The activation of NF‐κB is regulated by three major steps: phosphorylation of IκB, degradation of IκB and nuclear translocation of NF‐κB 29. Therefore, measurement of the presence of NF‐κB can indirectly reflect the activity of the transcription factor. Using Western blotting and immunohistochemistry, our results indicate that DCS significantly inhibits IκB and the phosphorylation of NF‐κB in NCI‐H460 cells and tumours. It has been previously reported that an increased NF‐κB expression is accompanied by an increased secretion of TNF‐α, IL‐1 and IL‐6 30. In the present study, DCS only inhibited the level of IL‐1, but had no effect on TNF‐α and IL‐6 in tumours by ELISA. The p38 pathway is also associated with the production of COX‐2, which is an important inflammation factor that catalyses prostaglandin synthesis 31. We show that DCS inhibits the expression of COX‐2, but has no effect on PEGES2 in NCI‐H460 cells and tumours based on Western blotting and immunohistochemistry. Therefore, inhibition of p38 activation by DCS appears to be an inducible factor of IL‐1 and COX‐2 down‐regulation in our model system.

To verify the mechanisms of DCS activity, microarray analysis was used to screen systematically the differentially expressed genes between control and DCS‐treated cells. The major findings of the microarray analyses were that the differentially expressed genes in DCS‐treated cells were associated with the cell cycle process. JNK and p38 MAPK signalling pathways are well‐known mediators of growth factor‐dependent cell survival, and tumour cell proliferation is closely related to the cell cycle. Cell cycle progression is closely related to various cyclins, cyclin‐dependent kinases (CDKs), CDK inhibitors and certain tumour suppressor gene products. Cell cycle regulation is downstream of JNK and p38 MAPKs, and genes closely related to the cell cycle, including cyclin D1, cyclin B1, CDK1, cyclin E1, cyclin A2 and CDK2, were confirmed to be altered using Western blotting. Meanwhile, our data showed that DCS‐treated NCI‐H460 cells were arrested in G1 phase, which accompanied by a decline in the levels of cyclin E and an increase in the levels of cyclin D1, cyclin B1 and CDK1. Flow cytometry analysis results demonstrated that DCS triggered NCI‐H460 cell apoptosis. These results are consistent with the results of our signalling pathway analyses.

Taken together, the findings reported in this work show that dihydroberberine increases the sensitivity of lung carcinoma to sunitinib. DCS shows more inhibition on lung cancer cell growth *in vitro* and *in vivo*, possibly by down‐regulating p38 and JNK MAPK signalling molecules, while simultaneously inhibiting IκB, NF‐κB phosphorylation and pro‐inflammatory cytokines. Inhibition of p38 activation by DCS could be responsible for IL‐1 and COX‐2 down‐regulation. Regulation of the cell cycle could subsequently lead to down‐regulation of JNK and p38, and further reduce IL‐1 secretion. This is the first evidence that the combination of dihydroberberine and sunitinib could be a new strategy for lung cancer treatment, and it may become a potential drug candidate.

## Conflict of interest

The authors declare no conflict of interest.

## Supporting information


**Fig. S1** Effects of dihydroberberine and sunitinib on the growth of different lung cancer cells. (**A**) Dihydroberberine (**B**) Sunitinib. NCI‐H460, A549, and NCI‐H1299 cell viabilities were determined by MTT assays in cells exposed to increasing concentrations of dihydroberberine and sunitinib for 48 hrs.

**Fig. S2** Effects of dihydroberberine combined with sunitinib at different concentrations on the growth of NCI‐H460 cells. Sunitinib were at 0, 1, 2, 4 μmol/l.(**A**) Dihydroberberine at 0 μmol/l. (**B**) Dihydroberberine at 6.25 μmol/l. (**C**) Dihydroberberine at 12.5 μmol/l. (**D**) Dihydroberberine at 25 μmol/l.

**Fig. S3** Effect of DCS on PI3K/Akt/mTOR signalling pathway proteins in NCI‐H460 cells. (**A**) Effect of DCS on the PI3K subunit protein expression of p110α, p110β, p110γ, p110III, p‐p85/p55, and p85 in NCI‐H460 cells and tumour tissues using Western blotting. (**B**,** C**) Quantification of the data from part (A). DCS had no effect on the PI3K subunits. (**D**) Effect of DCS on PI3K/Akt/mTOR signalling pathway protein expression of p‐AKT, AKT, p‐mTOR and mTOR in NCI‐H460 cells and tumour tissues using Western blotting. (**E**,** F**) Quantification of the data from part (D). DCS had no effect on PI3K/Akt/mTOR signalling pathway proteins.

**Fig. S4** The GO category for differentially expressed genes. (**A**) ‐logPvalue ≥ 2 was used as a cut‐off threshold to select significant GO categories. (**A**) The down‐regulated genes in the dihydroberberine‐treated group compared with the control group. (**B)** The up‐regulated genes in the dihydroberberine‐treated group compared with the control group. (**C**) The down‐regulated genes in the sunitinib‐treated group compared with the control group. (**D**) The up‐regulated genes in the sunitinib‐treated group compared with the control group.

**Fig. S5** Effects of dihydroberberine and/or sunitinib on cell apoptosis. Annexin V‐PI staining for apoptosis in NCI‐H460 cells treated with dihydroberberine and/or sunitinib.

**Table S1** CIs of combination treatment.
