# Supplementary figures and images for: Dihydroberberine exhibits synergistic effects with sunitinib on NSCLC NCI‐H460 cells by repressing MAP kinase pathways and inflammatory mediators
Source: J Cell Mol Med. 2017 Apr 26;21(10):2573–85. doi: 10.1111/jcmm.13178 (PMC5618684; doi:10.1111/jcmm.13178)

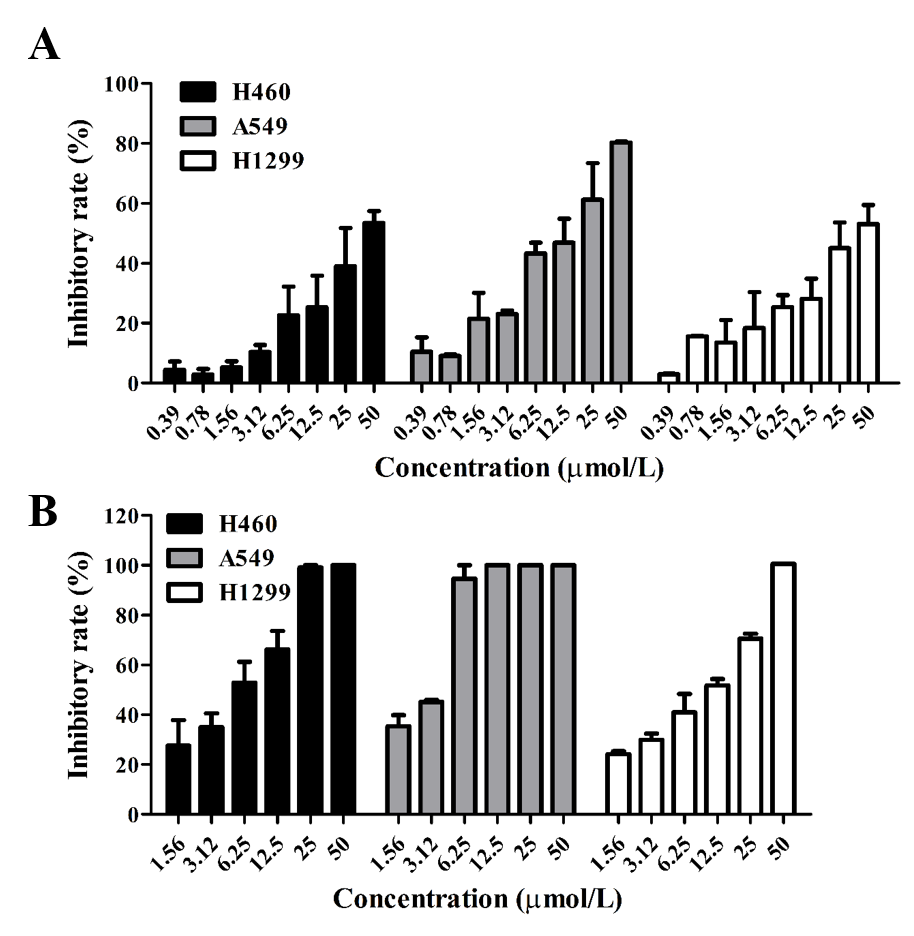

Supplement: Supplementary file 1 — Fig. S1 Effects of dihydroberberine and sunitinib on the growth of different lung cancer cells. (A) Dihydroberberine (B) Sunitinib. NCI‐H460, A549, and NCI‐H1299 cell viabilities were determined by MTT assays in cells exposed to increasing concentrations of dihydroberberine and sunitinib for 48 hrs. [file JCMM-21-2573-s001.tif]

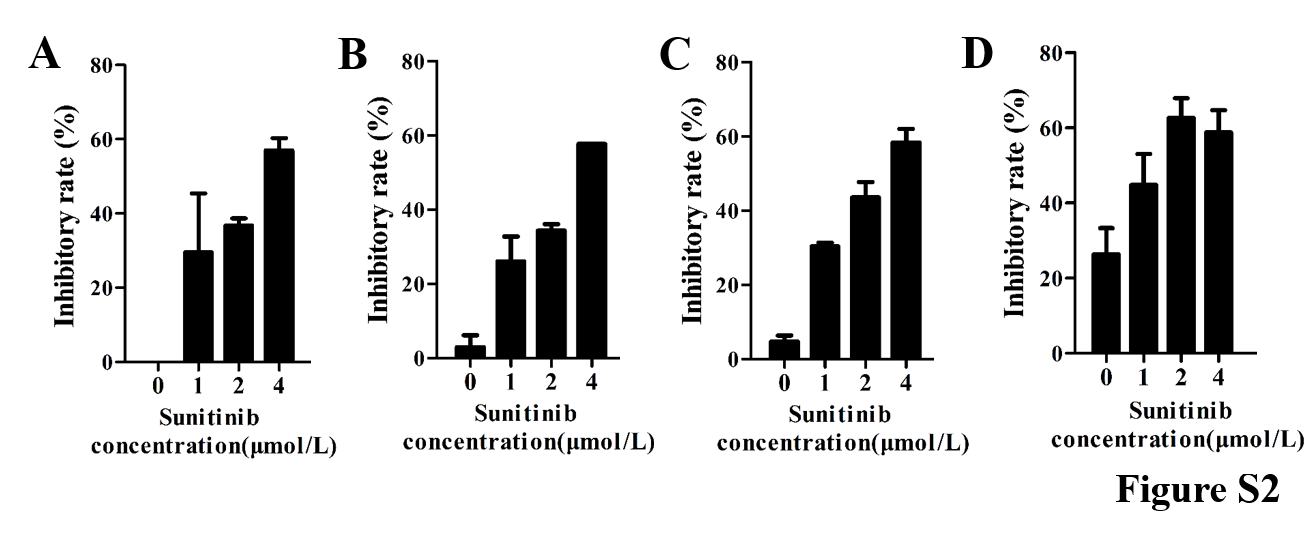

Supplement: Supplementary file 2 — Fig. S2 Effects of dihydroberberine combined with sunitinib at different concentrations on the growth of NCI‐H460 cells. Sunitinib were at 0, 1, 2, 4 μmol/l.(A) Dihydroberberine at 0 μmol/l. (B) Dihydroberberine at 6.25 μmol/l. (C) Dihydroberberine at 12.5 μmol/l. (D) Dihydroberberine at 25 μmol/l. [file JCMM-21-2573-s002.tif]

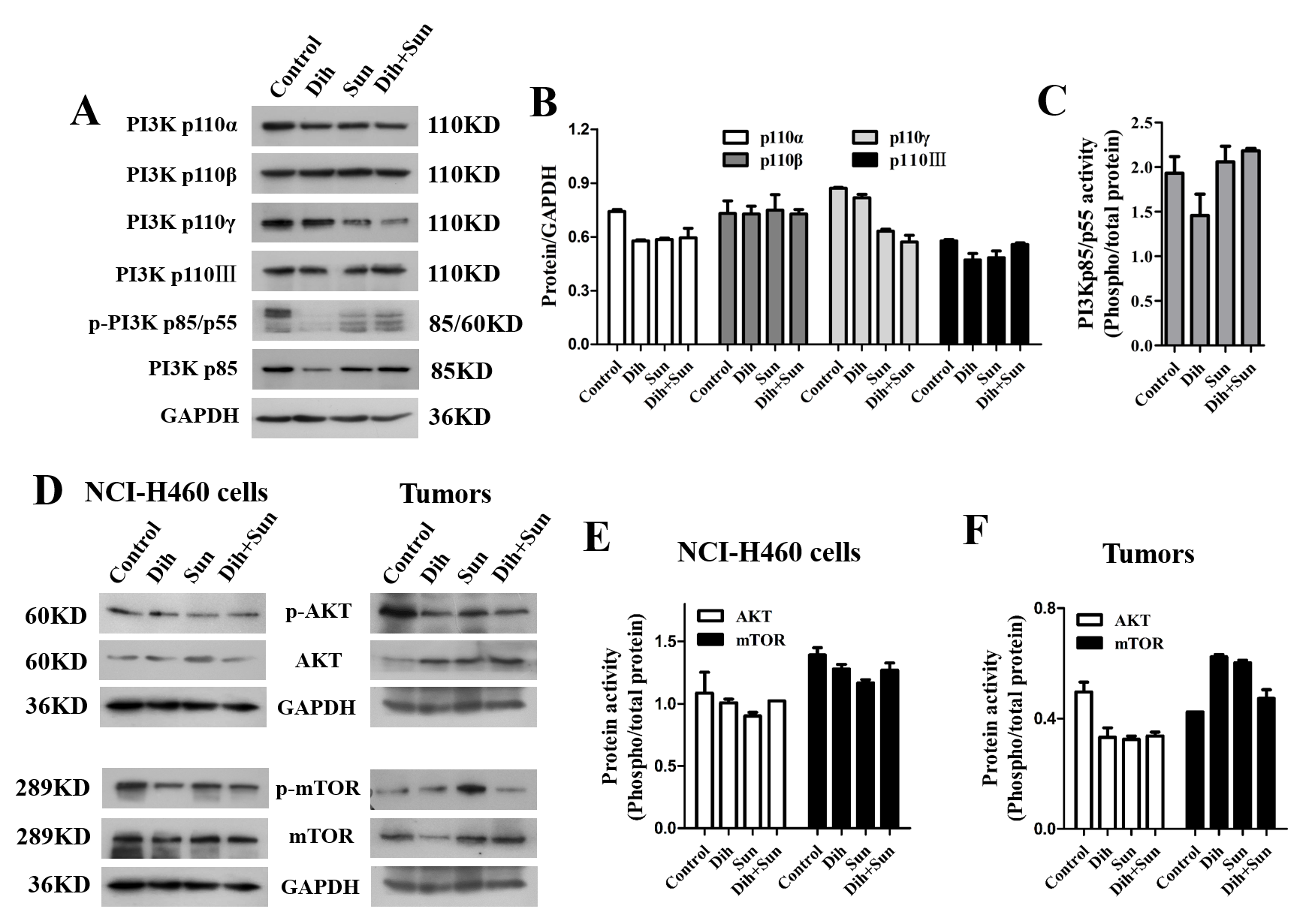

Supplement: Supplementary file 3 — Fig. S3 Effect of DCS on PI3K/Akt/mTOR signalling pathway proteins in NCI‐H460 cells. (A) Effect of DCS on the PI3K subunit protein expression of p110α, p110β, p110γ, p110III, p‐p85/p55, and p85 in NCI‐H460 cells and tumour tissues using Western blotting. (B, C) Quantification of the data from part (A). DCS had no effect on the PI3K subunits. (D) Effect of DCS on PI3K/Akt/mTOR signalling pathway protein expression of p‐AKT, AKT, p‐mTOR and mTOR in NCI‐H460 cells and tumour tissues using Western blotting. (E, F) Quantification of the data from part (D). DCS had no effect on PI3K/Akt/mTOR signalling pathway proteins. [file JCMM-21-2573-s003.tif]

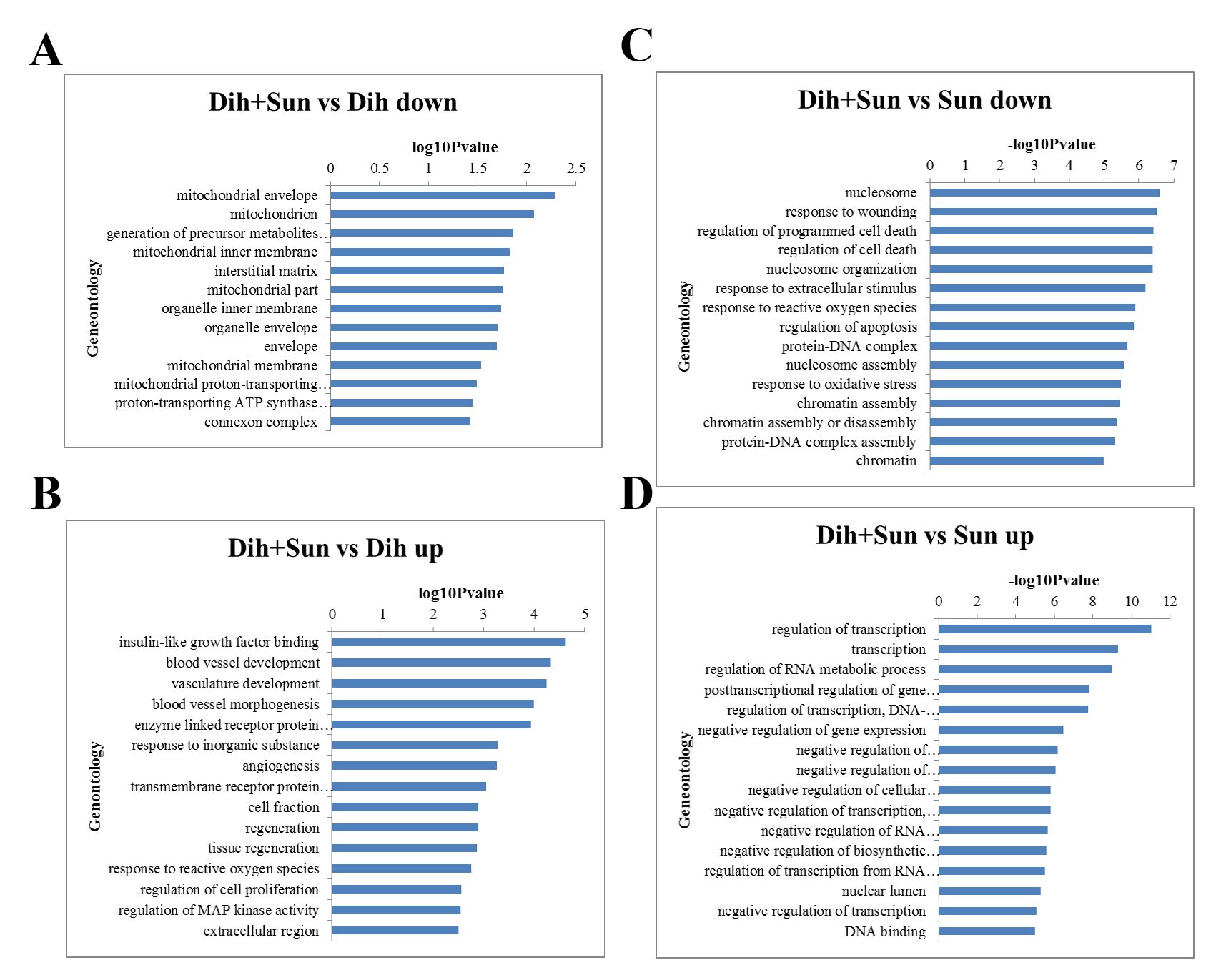

Supplement: Supplementary file 4 — Fig. S4 The GO category for differentially expressed genes. (A) ‐logPvalue ≥ 2 was used as a cut‐off threshold to select significant GO categories. (A) The down‐regulated genes in the dihydroberberine‐treated group compared with the control group. (B) The up‐regulated genes in the dihydroberberine‐treated group compared with the control group. (C) The down‐regulated genes in the sunitinib‐treated group compared with the control group. (D) The up‐regulated genes in the sunitinib‐treated group compared with the control group. [file JCMM-21-2573-s004.tif]

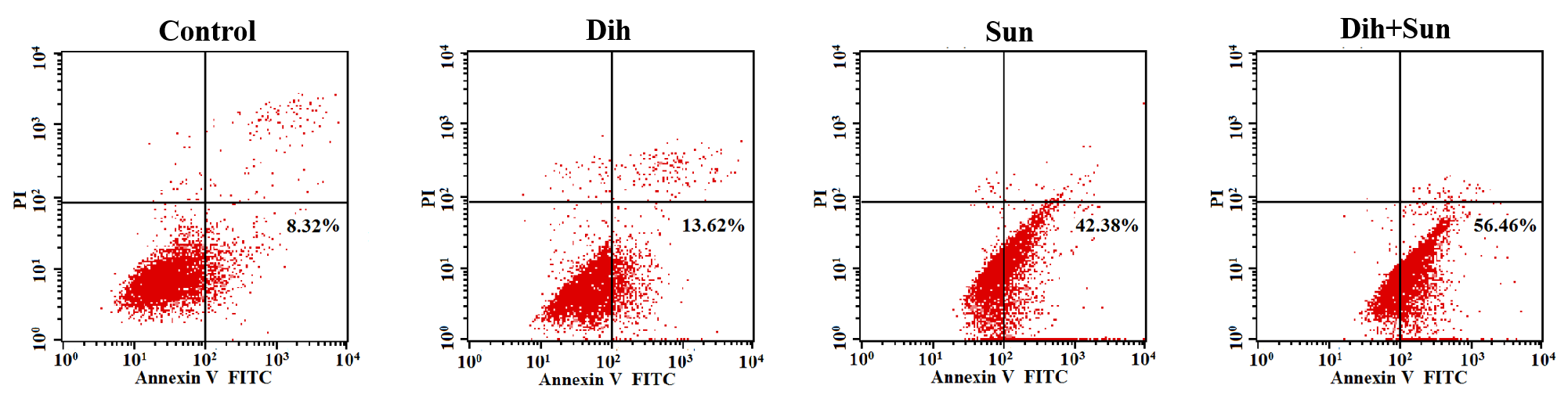

Supplement: Supplementary file 5 — Fig. S5 Effects of dihydroberberine and/or sunitinib on cell apoptosis. Annexin V‐PI staining for apoptosis in NCI‐H460 cells treated with dihydroberberine and/or sunitinib. [file JCMM-21-2573-s005.tif]
