# Supplementary material for: Dihydroberberine exhibits synergistic effects with sunitinib on NSCLC NCI‐H460 cells by repressing MAP kinase pathways and inflammatory mediators
Source: J Cell Mol Med. 2017 Apr 26;21(10):2573–85. doi: 10.1111/jcmm.13178 (PMC5618684; doi:10.1111/jcmm.13178)
Supplement: Supplementary file 6 — Table S1 CIs of combination treatment. [file JCMM-21-2573-s006.doc]

**Supplementary information**

**Supplementary Table. 1**

Table 1**.** CIs of combination treatment.

| Sunitinib  （μmol/L） | [Dihydroberberine](http://dj.iciba.com/二氢小檗碱-1.html)（μmol/L） | | |
| --- | --- | --- | --- |
|  | 25 | 12.5 | 6.25 |
| 4 | 0.86 | 0.99 | 0.99 |
| 2 | 1.17 | 1.10 | 0.89 |
| 1 | 0.93 | 0.92 | 0.83 |

CIs determine the type of interaction [Additive (0.85 ≤ Q ≤ 1.15); Synergistic (Q>1.15); Antagonistic (CI<0.85)].
